# Comparing the Performance of Machine Learning Models and Conventional Risk Scores for Predicting Major Adverse Cardiovascular Cerebrovascular Events After Percutaneous Coronary Intervention in Patients With Acute Myocardial Infarction: Systematic Review and Meta-Analysis

**DOI:** 10.2196/76215

**Published:** 2025-07-18

**Authors:** Min-Young Yu, Hae Young Yoo, Ga In Han, Eun-Jung Kim, Youn-Jung Son

**Affiliations:** 1 Graduate School of Nursing, Chung-Ang University, Seoul, Republic of Korea; 2 Red-Cross College of Nursing, Chung-Ang University, 84 Heukseokro, Dongjak gu, Seoul, 06974, Republic of Korea, 82 2-820-5198

**Keywords:** machine learning, mortality, myocardial infarction, patient readmission, percutaneous coronary intervention, prediction algorithm, statistical model

## Abstract

**Background:**

Machine learning (ML) models may offer greater clinical utility than conventional risk scores, such as the Thrombolysis in Myocardial Infarction (TIMI) and Global Registry of Acute Coronary Events (GRACE) risk scores. However, there is a lack of knowledge on whether ML or traditional models are better at predicting the risk of major adverse cardiovascular and cerebrovascular events (MACCEs) in patients with acute myocardial infarction (AMI) who have undergone percutaneous coronary interventions (PCI).

**Objective:**

The aim of this study is to systematically review and critically appraise studies comparing the performance of ML models and conventional risk scores for predicting MACCEs in patients with AMI who have undergone PCI.

**Methods:**

Nine academic and electronic databases including PubMed, CINAHL, Embase, Web of Science, Scopus, ACM, IEEE, Cochrane, and Google Scholar were systematically searched from January 1, 2010, to December 31, 2024. We included studies of patients with AMI who underwent PCI, and predicted MACCE risk using ML algorithms or conventional risk scores. We excluded conference abstracts, gray literature, reviews, case reports, editorials, qualitative studies, secondary data analyses, and non-English publications. Our systematic search yielded 10 retrospective studies, with a total sample size of 89,702 individuals. Three validation tools were used to assess the validity of the published prediction models. Most included studies were assessed as having a low overall risk of bias.

**Results:**

The most frequently used ML algorithms were random forest (n=8) and logistic regression (n=6), while the most used conventional risk scores were GRACE (n=8) and TIMI (n=4). The most common MACCEs component was 1-year mortality (n=3), followed by 30-day mortality (n=2) and in-hospital mortality (n=2). Our meta-analysis demonstrated that ML-based models (area under the receiver operating characteristic curve: 0.88, 95% CI 0.86‐0.90; *I²*=97.8%; *P*<.001) outperformed conventional risk scores (area under the receiver operating characteristic curve: 0.79, 95% CI 0.75‐0.84; *I²*=99.6%; *P*<.001) in predicting mortality risk among patients with AMI who underwent PCI. Heterogeneity across studies was high. Publication bias was assessed using a funnel plot. The top-ranked predictors of mortality in both ML and conventional risk scores were age, systolic blood pressure, and Killip class.

**Conclusions:**

This review demonstrated that ML-based models had superior discriminatory performance compared to conventional risk scores for predicting MACCEs in patients with AMI who had undergone PCI. The most commonly used predictors were confined to nonmodifiable clinical characteristics. Therefore, health care professionals should understand the advantages and limitations of ML algorithms and conventional risk scores before applying them in clinical practice. We highlight the importance of incorporating modifiable factors—including psychosocial and behavioral variables—into prediction models for MACCEs following PCI in patients with AMI. In addition, further multicenter prospective studies with external validation are required to address validation limitations.

## Introduction

Acute myocardial infarction (AMI) is associated with a greater risk of mortality due to sustained ischemia and necrosis of the myocardium, requiring aggressive treatment [1]. Percutaneous coronary intervention (PCI) can promptly reopen infarcted blood vessels and restore reperfusion in AMI patients [2]. However, the risk of restenosis within 1 year after successful PCI remains high at around 10%‐20% [3], potentially resulting in adverse health outcomes and increasing health care costs [2]. Notably, major adverse cardiovascular and cerebrovascular events (MACCEs) are an important issue in managing patients with AMI undergoing PCI [3]. MACCEs encompass composite outcomes such as cardiovascular-related death, hospitalization for unstable angina or heart failure, recurrent myocardial infarction, stroke, and coronary revascularization, including PCI and coronary artery bypass grafting [3]. Therefore, promptly assessing high-risk patients and reducing the risk of MACCEs is crucial for quality of care.

Conventional risk scores (CRS), such as the Thrombolysis in Myocardial Infarction (TIMI) and Global Registry of Acute Coronary Events (GRACE) scores, are commonly used for predicting MACCEs due to their long-established reliability and ease of application in patients with cardiovascular diseases [4]. However, these conventional scores have limitations, as they cannot capture the complex interplay of patient-specific characteristics, particularly nonlinear correlations within datasets [3,5].

Machine learning (ML)–based prediction models have recently gained traction, offering the ability to detect subtle patterns that conventional GRACE and TIMI scores, which rely on logistic regression, may overlook in patients with AMI [5,6]. However, achieving higher accuracy with ML-based models requires large datasets and has the disadvantage of limited ability to clarify causal relationships between variables [7]. Therefore, comparing the performance of ML-based models with CRS is essential to elucidate their similarities and differences in risk prediction [8].

Several systematic reviews have examined ML-based models for predicting adverse cardiac events after PCI [3,5], MACCEs in acute coronary syndrome, or outcomes in older adults who have undergone PCI [9]. These reviews focused on ML and conventional statistical models for predicting negative health outcomes in individuals with coronary artery disease, both with and without PCI. Only 2 reviews have compared the performance of ML-based models with conventional statistical models for predicting readmission or mortality after MI [6,10]. However, these reviews did not focus on predicting MACCEs after PCI in AMI patients.

This study aimed to appraise studies comparing the performance of ML-based models with CRS in predicting MACCEs after PCI in AMI patients. Furthermore, this study identifies common risk factors for mortality after PCI using both ML and CRS, which may aid in developing clinical guidelines and improving personalized risk assessment.

## Methods

### Study Design

This systematic review adhered to the Checklist for Critical Appraisal and Data Extraction for Systematic Reviews of Prediction Modelling Studies (CHARMS) [11] and the PRISMA (Preferred Reporting Items for Systematic Reviews and Meta-Analyses) [12] guidelines. The PRISMA checklist is available in Checklist 1. This review protocol was registered in the International Prospective Register of Systematic Reviews (PROSPERO) under the identifier CRD42024557418.

### Data Sources and Search Strategy

A comprehensive search was performed in multiple databases (PubMed, CINAHL, Embase, Web of Science, Scopus, ACM, IEEE, Cochrane, and Google Scholar), focusing on literature published between January 1, 2010, and December 31, 2024. The search strategy incorporated the use of both Medical Subject Headings (MeSH) and free-text terms related to acute coronary syndrome, AMI, PCI, MACCEs, machine learning, mortality, readmission, and prediction models. Detailed information on the search terms is shown in Multimedia Appendix 1.

### Study Selection and Data Extraction

EndNote 20 (Clarivate Plc) and Microsoft Excel 2019 (Microsoft Corp) were used to retrieve the full text of all retrieved peer-reviewed articles, remove duplicates, and manage the screening process. After applying these keyword-based filters and database classifications, 75,122 records were exported to EndNote 20 for further review. Duplicate references were then removed using both manual review and automated tools via EndNote 20 and Microsoft Excel 2019. Manual title and abstract screening (n=44,559) were then conducted by 2 independent reviewers (M-YY and GIH) based on predefined eligibility criteria. Two independent reviewers (M-YY and GIH) evaluated all potentially relevant full texts (n=444). We resolved discrepancies between reviewers through discussion or by involving a third reviewer (Y-JS) until a consensus was reached. Two authors (M-YY and GIH) performed data extraction, which was subsequently verified by a third author (Y-JS).

PICO (participant, intervention, comparison, and outcomes) was used to build eligibility criteria (Table 1). We included studies that met the following criteria: (1) involved adult patients (18 y or older) diagnosed with AMI, including ST-segment elevation myocardial infarction and non–ST-segment elevation myocardial infarction; (2) included patients who underwent PCI; and (3) predicted MACCEs risk using ML algorithms and CRS with statistical methods. We excluded studies focused on confirming PCI procedure outcomes and studies that solely compared ML model performance. In addition, to ensure the consistent and accurate interpretation of machine learning terminology and research methodologies, while minimizing potential translation bias, the search was restricted to English-language publications. The publication period was restricted to studies published between 2010 and 2024 to capture the full range of research from the early emergence of clinical machine learning predictive models to the most recent advancements. To ensure consistent study selection and reduce potential bias, inclusion and exclusion criteria were applied systematically for study designs and publication types.

**Table 1.** Eligibility criteria for the systematic review and meta-analysis using the PICO (participant, intervention, comparison, and outcomes) format.

Item	Inclusion criteria	Exclusion criteria
Participants	Studies involving adult patients (≥18 y old) diagnosed with AMI^a^_,_ including STEMI^b^ and NSTEMI^c^	Studies involving children and adolescents (<18 y old) Studies focused exclusively on specific subpopulations (eg, only women) Studies involving patients with tumors, severe infections, or trauma, or those recovering from acute infections
Intervention	Studies involving patients who underwent percutaneous coronary intervention	Studies evaluating only the performance outcomes of percutaneous coronary intervention procedures Studies involving combination therapies such as coronary artery bypass grafting, thrombolytics, and medical therapy
Comparison	Studies that predicted MACCEs^d^ risk using ML^e^ algorithms (defined if the study authors reported an ML algorithm) and conventional risk scores (traditional statistical methods)	Studies comparing model performance without identifying predictors of MACE^f^/ MACCEs in multivariable analysis
Outcomes	Studies that predicted MACCEs	Studies that assessed only mortality as an endpoint in validating MACE/MACCEs outcomes
Language	English papers	Non-English papers
Publication period	Published between 2010 and 2024	Studies published before 2010 and after 2024
Study designs	Original quantitative studies (randomized control trials, cohort, cross-sectional, etc)	Reviews or meta-analyses, case reports/series and editorials, qualitative studies, secondary data analyses, basic physiology studies Studies conducted on nonhuman participants
Publication types	Full published original research papers or journal articles	Conference abstracts, dissertations and theses, editorials Duplicated studies or informal publications Retracted articles due to ethical or data integrity issues Articles for which the full text was not available

a AMI: acute myocardial infarction.

b STEMI: ST-segment elevation myocardial infarction.

c NSTEMI: non–ST-segment elevation myocardial infarction.

d MACCEs: major adverse cardiovascular and cerebrovascular events.

e ML: machine learning.

f MACE: major adverse cardiovascular event.

### Quality Appraisals and Risk of Bias Assessment

In our review, the completeness and reliability of the analyzed literature were assessed using 3 tools: the Transparent Reporting of a Multivariable Prediction Model for Individual Prognosis or Diagnosis + AI (TRIPOD + AI) [13], the Prediction Model Risk of Bias Assessment Tool (PROBAST) [14], and the CHARMS [11].

TRIPOD + AI is a checklist that outlines essential items for effectively reporting studies that develop or evaluate prediction models using ML or statistical approaches. The checklist consists of 27 primary items covering various sections: title, abstract, introduction, methods, open science practices, patient and public involvement, results, and discussion [13]. Compliance with the TRIPOD + AI checklist was evaluated by scoring each item 1 point if reported and 0 points if not reported. The risk of bias and clinical applicability of each included study was evaluated using the PROBAST, which comprises 4 domains and 20 signal questions to evaluate bias and applicability [14]. Lastly, the CHARMS was also adopted [15].

### Data Synthesis and Analysis

Data extraction was done by 3 reviewers independently. It included details such as the first author’s name, year of publication, country, sample size, study design, study characteristics (eg, ML algorithm and type of CRS), outcome measurement, predictors, and their performance. This review reported the area under the receiver operating characteristic curve (AUROC) as a measure of predictive accuracy and ML performance [16]. The AUROC values were reported with 95% CIs.

Low, moderate, and high heterogeneity were considered when the value of the *I²* statistic was 25%, 50%, and 75%, respectively [17]. A random-effects model was used for the analysis to address heterogeneity in the pooled results and appropriately integrate effect sizes, using the inverse variance method for pooling [18]. To assess publication bias, a funnel plot was generated for visual inspection of asymmetry. Egger’s regression test [19] and Begg’s test [20] were conducted to further evaluate the presence of potential bias. Additionally, the trim-and-fill method [21] was applied to adjust for any bias detected by the funnel plot. Statistical analyses were performed using the “metagen” function in the “meta” package of R 4.2.2 (R Foundation for Statistical Computing) [22,23].

### Ethical Considerations

The institutional review board of Chung-Ang University (number 1041078‐20240611-HR-144) approved the study protocol.

## Results

### Characteristics of the Included Studies

**Figure 1.**
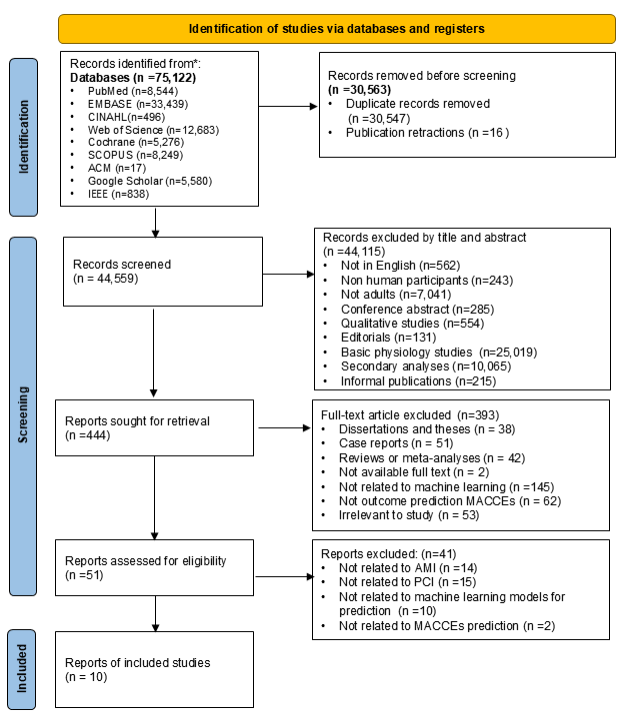
PRISMA flow diagram of study selection. AMI: acute myocardial infarction; MACCEs: major adverse cardiac and cerebrovascular events; PCI: percutaneous coronary intervention; PRISMA: Preferred Reporting Items for Systematic Reviews and Meta-Analyses.

We identified 75,122 records through a comprehensive search strategy, as presented in the PRISMA 2020 flowchart (Figure 1). After removing 30,547 duplicate records, 44,575 records remained for screening. Of these, 16 were excluded as they had been retracted due to issues such as ethical concerns or compromised data integrity. Subsequently, 44,559 records were screened by title and abstract, resulting in the selection of 444 full-text articles for further evaluation. Following full-text screening, an additional 393 articles were excluded for the following reasons: dissertations and theses (n=38); case reports (n=51); reviews or meta-analyses (n=42); full text not available (n=2); not related to machine learning (n=145); not focused on predicting MACCEs (n=62); and irrelevant to the study objective (n=53).

In addition, conference abstracts (n=285) corresponding to gray literature were classified through title and abstract verification, while dissertations and theses (n=38) were distinguished through full-text article verification. Conference abstracts and dissertations/theses were considered gray literature. Conference abstracts were excluded due to the lack of full methodological information and peer review, while dissertations were excluded depending on full-text availability and the ability to assess methodological quality.

Ultimately, 10 articles [24-33] comprising 89,702 samples were included in this review (Table 2). All articles were published between 2017 [24] and 2024 [32,33]. Seven studies originated from Asian countries: 3 from Korea [25-27], 3 from China [29,31,32], and 1 from Malaysia [28]. All studies were retrospective, with sample sizes for model development ranging from 466 [31] to 22,875 [26]. Six studies used data from multicenter or national registries.

**Table 2.** Characteristics of studies included in this review (N=10).

First author	Year	Country	Data source	Study design	Sample size (n)	ML^a^ algorithms	Conventional risk score	Outcome variables	Main findings
Shouval et al [24]	2017	Israel	2006‐2013, multicenter registry	Retrospective	2782	RF^b^, LR^c^, NB^d^, ADT^e^, PART^f^, AdaBoost^g^	TIMI^h^ and GRACE^i^	30-day mortality	RF, NB, and AdaBoost models showed similar performance to GRACE, and all models outperformed TIMI.
Kim et al [25]	2022	Korea	2005‐2008, multicenter registry	Retrospective	10,813	DNN^j^, GBM^k^, GLM^l^	GRACE	1-year MACCEs^m^	The ML model outperformed GRACE with an accuracy of over 95%.
Kwon et al [26]	2019	Korea	2008‐2013, multicenter registry	Retrospective	22,875	RF, LR, DNN	TIMI, GRACE, and ACTION^n^	1-year mortality	The ML model was 30.9% more accurate than GRACE.
Sherazi et al [27]	2020	Korea	2005‐2008, multicenter registry	Retrospective	8227	RF, GBM, GLM, DNN	GRACE	1-year mortality	The performance of the ML model improved by an average of 0.08 compared to GRACE.
Aziz et al [28]	2021	Malaysia	2006‐2016, national registry	Retrospective	12,368	RF, LR, SVM^o^	TIMI	In-hospital mortality	The ML model outperformed TIMI.
Bai et al [29]	2021	China	2016‐2020, EHR^p^ from single hospital	Retrospective	656	RF, LR, KNN^q^, CatBoost, XGBoost^r^	GRACE	1-year mortality	CatBoost model outperformed GRACE.
Hadanny et al [30]	2021	Israel, England, and Wales	2006‐2016, multicenter registry	Retrospective	25,475	RF	GRACE	30-day mortality	The RF model showed higher discriminatory power than GRACE.
Fang et al [31]	2022	China	2018‐2022, EHR from single hospital	Retrospective	466	LR	TIMI	In-hospital MACCEs	The LR model predicted higher performance than TIMI.
Liu et al [32]	2024	China	2014‐2022, EHR from single hospital	Retrospective	1363	RF, LR, DT^s^, SVM, XGBoost, AdaBoost	GRACE, KAMIR^t^, and ACEF^u^	1-year readmission	The LR model showed the best performance.
Shakhgeldyan et al [33]	2024	Russia	2015‐2021, EHR from single hospital	Retrospective	4677	RF, MLR^v^, SGB^w^	GRACE	In-hospital mortality	The model built with important variables showed the highest accuracy.

a ML: machine learning.

b RF: random forest.

c LR: logistic regression.

d NB: naïve Bayes.

e ADT: alternating decision trees.

f PART: pruned rules from classification trees.

g AdaBoost: adaptive boosting.

h TIMI: thrombolysis in myocardial infarction.

i GRACE: Global Registry of Acute Cardiac Events.

j DNN: deep neural network.

k GBM: gradient boosting machine.

l GLM: generalized linear model.

m MACCEs: major cardiovascular and cerebrovascular adverse events.

n ACTION: Acute Coronary Treatment and Intervention Outcomes Network scores.

o SVM: support vector machine.

p EHR: electronic health record.

q KNN: k-nearest neighbor.

r XGBoost: extreme gradient boosting.

s DT: decision tree.

t KAMIR: Korea Acute Myocardial Infarction Registry.

u ACEF: age, creatinine, and ejection fraction score.

v MLR: multinomial logistic regression.

w SGB: stochastic gradient boosting.

The most frequently used ML-based models were random forest (RF; n=8) and logistic regression (LR; n=6), while the most used CRS were GRACE (n=8) and TIMI (n=4). The most common MACCEs components were 1-year mortality (n=3), followed by 30-day mortality (n=2) and in-hospital mortality (n=2). All studies included in this review reported that ML-based models outperformed CRS, including GRACE and TIMI, demonstrating higher accuracy and discriminatory power.

### Risk of Bias and Applicability of Studies

We critically appraised the 10 studies included in the review (Multimedia Appendix 2). The overall quality of the studies, as assessed using the TRIPOD + AI guidelines, showed that only 4 studies met more than 70% of the TRIPOD + AI criteria. Open science practices, which are included as key components of the TRIPOD + AI guidelines, are vital to health care prediction model research as they foster transparency, reproducibility, and interdisciplinary collaboration [13]. However, only 2 studies [32,33] provided accessible and verifiable protocols.

The PROBAST checklist was used to evaluate the quality of the included studies, specifically assessing the risk of bias and applicability concerns. According to the PROBAST checklist, the overall risk of bias was low in most studies included in this review. However, potential bias was identified in the outcome definition [31], and low applicability was observed due to unclear reporting and methodological concerns [25-27]. In the participant domain, all studies were rated as having a low risk of bias. In contrast, in the applicability domain, 2 studies [31,32] were identified as of high concern due to the inclusion of narrowly selected populations, which may not be representative of the intended target population.

Based on the CHARMS, 2 studies exhibited a high risk of bias in at least 2 major categories [26,31]. Although all the studies demonstrated low risk of bias in most domains—including data source, participant selection, and outcome—attrition-related bias was common, with 7 studies failing to report participant losses or methods for handling missing data.

### Model Performance of Risk Prediction for MACCEs

Table 3 and Table 4 present the comparative performance of ML-based models and CRS in terms of accuracy and validation methods, respectively. If a study applied multiple ML-based models on the same dataset, the model with the highest predictive performance—based on accuracy or other relevant metrics—was selected for analysis to avoid unit-of-analysis errors. All performance metrics are rounded to 2 decimal places for consistency.

As shown in Table 3, ML-based models used between 13 [26] and 136 [33] candidate variables. Only 2 studies conducted external validation [26,30], and 2 assessed model calibration [30,31]. The AUROC values for the best-performing ML-based models ranged from 0.77 (95% CI 0.74‐0.79) for ML-LR [32] to 0.99 for SMOTEENN-CatBoost [29]. Table 4 summarizes the performance of the CRS models, which included between 4 [32] and 23 [24] significant variables. GRACE was the most commonly used CRS model (n=8), with AUROC values ranging from 0.65 [32] to 0.88 [30].

**Table 3.** The performance of machine learning models for the prediction of major adverse cardiac and cerebrovascular events (N=10).

Reference	Candidate variables (n)	Validation method	External validation	Calibration	Machine learning model performance		
Best model	AUROC^a^	95% CI
[23]	54	10-fold	No	Not reported	RF^b^	0.91	0.84‐0.91
[24]	51	10-fold	No	Not reported	DNN^c^	0.97	Not reported
[25]	13	Not reported	Yes	Not reported	DAMI^d^	0.91	0.90‐0.91
[26]	69	4-fold	No	Not reported	DNN	0.90	Not reported
[27]	50	10-fold	No	Not reported	SVMvarImp-SBE-SVM^e^	0.88	0.85‐0.91
[28]	41	10-fold	No	Not reported	SMOTEEN^f^-CatBoost	0.99	Not reported
[29]	32	Not reported	Yes	Yes	RF	0.91	0.89‐0.93
[30]	39	10-fold	No	Yes	Nomogram	0.83	0.79‐0.87
[31]	96	Not reported	No	Not reported	ML-LR^g^	0.77	0.74‐0.79
[32]	136	10-fold	No	Not reported	MLR^h^	0.90	0.84‐0.96

a AUROC: area under the receiver operating characteristic curve.

b RF: random forest.

c DNN: deep neural network.

d DAMI: deep-learning-based risk stratification for the mortality of patients with acute myocardial infarction.

e SVMvarImp-SBE-SVM: support vector machine variable importance with sequential backward elimination and support vector machine classifier.

f SMOTEENN: hybrid sampling algorithm of synthetic minority oversampling technique (SMOTE) and edited nearest neighbor (ENN) algorithms.

g ML-LR: constructed by logistic regression algorithm.

h MLR: multivariate logistic regression.

**Table 4.** The performance of conventional risk score models for the prediction of major adverse cardiac and cerebrovascular events (N=10).

Reference	Significant variable (n)	Conventional risk score model performance		
Model	AUROC^a^	95% CI
[23]	GRACE: 14, TIMI: 23	GRACE^b^, TIMI^c^	0.87, 0.82	Not reported
[24]	8	GRACE	0.76	Not reported
[25]	13	GRACE, TIMI, ACTION^d^	0.85, 0.78, 0.85	0.85‐0.86, 0.78‐0.79, 0.85‐0.86
[26]	9	GRACE	0.81	Not reported
[27]	9	TIMI	0.81	0.77‐0.80
[28]	Not reported	GRACE	0.80	Not reported
[29]	8	GRACE	0.88	0.87‐0.90
[30]	Not reported	TIMI	0.70	Not reported
[31]	4	GRACE	0.65	0.62‐0.67
[32]	5	GRACE	0.83	0.78‐0.89

a AUROC: area under the receiver operating characteristic curve.

b GRACE: Global Registry of Acute Cardiac Events.

c TIMI: Thrombolysis in Myocardial Infarction.

d ACTION: Acute Coronary Treatment and Intervention Outcomes Network scores.

### Common Predictors of MACCEs and Mortality in ML and CRS

Table 5 presents a detailed overview of the significant variables identified in both ML-based models and CRS models. The significant variables identified in ML and CRS models were categorized into 9 groups: sociodemographics, vital signs, electrocardiogram findings, laboratory findings, medical history, medication, angiographic findings, cardiac arrest, and others.

**Table 5.** Top-ranked predictors of studies included in this review.

Category	The most important predictors of MACCEs^a^ [study number]		Common predictors of mortality
Machine learning models	Conventional risk score model
Sociodemographics	Age [23,24,25,27,29,32], body mass index [25,26,27,29], gender [25,26,27], smoking history [23,24,26,27]	Age [23,24,25,30], weight [27]	Age [23,25]
Vital signs	SBP^b^ [23,25,27,29,32], DBP^c^ [23,27], MAP^d^ [29], heart rate [25,26,27,29,32]	SBP [24,25,27,30], DBP [27], MAP [29], heart rate [24,26,30]	SBP [25,27], DBP [27], MAP [29], heart rate [26]
Electrocardiogram findings	BBB^e^ [27], ST elevation [25,27]	BBB [30], ST elevation [24,30]	Not available
Laboratory findings	ALT^f^ [28], cholesterol [29], creatinine [23,24,25,26,29,32], CRP^g^ [25], CK-MB^h^ [25], eosinophils [32], glucose [23,25,26,27,29], hemoglobin [29], LDH^i^ [28], LDL^j^ [25,26], neutrophils [32], NT-proBNP^k^ [28,30], thrombocytes [32]	Creatinine [23, 24, 26, 30], CRP [31], glucose [24,25]	Creatinine [26], glucose [25]
Medical history	AHF^l^ [10], CHF^m^ [23], CKD^n^ [26], DM^o^ [24,26,27,28], family history of IHD^p^ [24], malignant neoplasm [26], Killip class [24,25,26,27,29,30,32]	CHF [27], hypertension [30], Killip class [24,25,26,30]	Killip class [25,26]
Medication	Aspirin [27], beta-blockers [27], diuretics [26,27], insulin [27], Mucomyst [26], statins [24,27]	Beta-blockers [27], oral hypoglycemic agent statins [27]	Beta-blockers [27], statins [27]
Angiographic findings	CABG^q^ [24], cardiac catheterization [27], time from onset to PCI^r^ [29]	2-vessel CAD^s^ [24]	Not available
Cardiac arrest	IHCA^t^ [24,30], OHCA^u^ [25]	IHCA [24,30], OHCA [25]	OHCA [25]
Others	Communication ability [31], discharge outcomes [31], early invasive therapy [26], nonweekday admissions [28]	Time to treatment [27]	Not available

a MACCEs: major cardiovascular and cerebrovascular adverse events.

b SBP: systolic blood pressure.

c DBP: diastolic blood pressure.

d MAP: mean arterial pressure.

e BBB: bundle branch block.

f ALT: alanine aminotransferase.

g CRP: c-reactive protein.

h CK-MB: creatine kinase myoglobin.

i LDH: lactate dehydrogenase.

j LDL: low density lipoprotein.

k NT-proBNP: N-terminal pro-brain natriuretic peptide.

l AHF: acute heart failure.

m CHF: chronic heart failure.

n CKD: chronic kidney disease.

o DM: diabetes mellitus.

p IHD: ischemic heart disease.

q CABG: coronary artery bypass grafting.

r PCI: percutaneous coronary intervention.

s CAD: coronary artery disease.

t IHCA: in-hospital cardiac arrest.

u OHCA: out-of-hospital cardiac arrest.

Our review identified 16 common predictors of MACCEs, including age, systolic blood pressure (SBP), diastolic blood pressure (DBP), mean arterial pressure (MAP), heart rate, bundle branch block and ST elevation (electrocardiogram findings), creatinine, C-reactive protein, glucose, chronic heart failure, Killip class, beta-blocker and statin use, and in-hospital and out-of-hospital cardiac arrest, all of which were significantly associated with a high risk of MACCEs in both ML-based models and CRS models.

Our review identified 10 common predictors of mortality in both models: age, SBP, DBP, MAP, heart rate, creatinine, glucose, Killip class, beta-blocker and statin use, and out-of-hospital cardiac arrest, all of which were related to a high risk of mortality. Age, SBP, and Killip class were the strongest predictors of mortality after PCI in patients with AMI (MultimediaAppendices 3  4).

### Meta-Analysis Results for Mortality Risk Prediction

**Figure 2.**
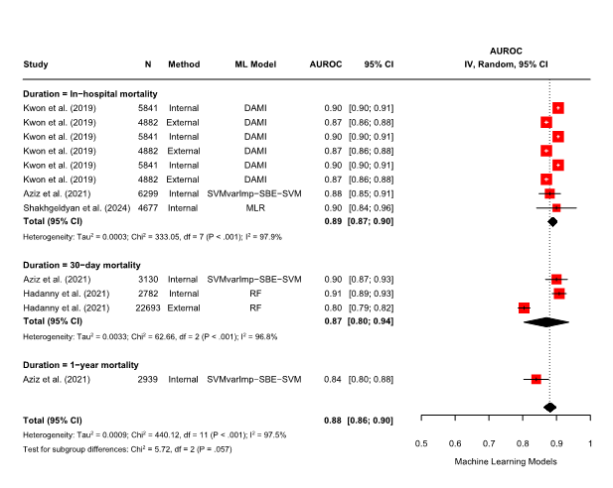
Forest plot of pooled AUROC estimates from a random-effects meta-analysis: machine learning models. AUROC: area under the receiver operating characteristic curve; DAMI: deep-learning–based risk stratification for the mortality of patients with acute myocardial infarction; ML: machine learning; MLR: multivariate logistic regression; RF: random forest; SVM: support vector machine; SVMvarImp-SBE-SVM: SVM variable importance with sequential backward elimination and SVM classifier.

This meta-analysis included 4 studies reporting AUROC values for mortality prediction, from which 12 AUROC values were obtained based on the validation method and mortality duration (Figures2  3).

**Figure 3.**
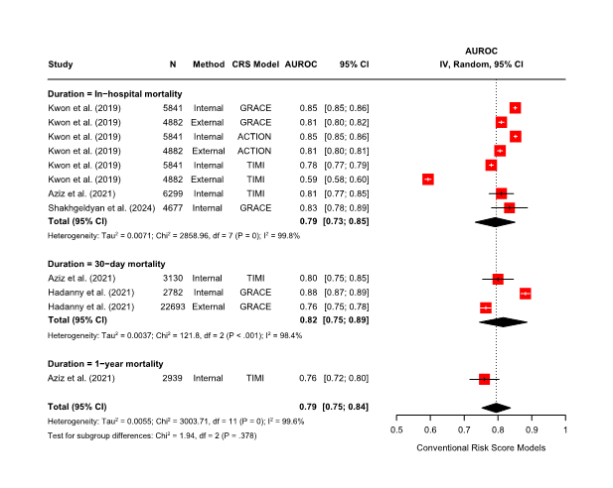
Forest plot of pooled AUROC estimates from a random-effects meta-analysis: conventional risk score models. ACTION: Acute Coronary Treatment and Intervention Outcomes Network scores; AUROC: area under the receiver operating characteristic curve; CRS: conventional risk score; GRACE: Global Registry of Acute Cardiac Events; TIMI: Thrombolysis in Myocardial Infarction.

Using a random-effects model, the meta-analysis demonstrated that the ML-based models (AUROC: 0.88, 95% CI 0.86‐0.90; *I²*=97.5%; *P*<.001) outperformed CRS models (AUROC: 0.79, 95% CI 0.75‐0.84; *I²*=99.6%; *P*<.001) in predicting the mortality risk of patients with AMI who had undergone PCI. Substantial heterogeneity was observed across studies. A subgroup meta-analysis was also conducted based on mortality duration. ML-based models outperformed CRS models in both in-hospital (AUROC: 0.89, 95% CI 0.87‐0.90; *I²*=97.9%; *P*<.001 vs AUROC: 0.79, 95% CI 0.73‐0.85; *I²*=99.8%; *P*<.001) and 30-day mortality prediction (AUROC: 0.87, 95% CI 0.80‐0.94; *I²*=96.8%, *P*<.001 vs AUROC: 0.82, 95% CI 0.75‐0.89; *I²*=98.4%, *P*<.001), with high heterogeneity across studies.

To statistically confirm whether this observed performance difference was significant, a meta-regression analysis was conducted with model type (ML-based vs CRS) as the moderator. The results showed that ML-based models had significantly higher AUROC values than CRS models (*β*=.09, 95% CI 0.04‐0.13; *P*<.001). To further explore sources of heterogeneity, a meta-regression analysis was conducted using log-transformed sample size as a moderator. This revealed a statistically significant negative association between sample size and AUROC for ML-based models (*β*=–.04, 95% CI –0.07 to 0.01; *P*=.02). In contrast, no significant association was observed for CRS models (*β*=–.02, 95% CI –0.10 to 0.07; *P*=.66).

To assess the reliability and validity of the included studies, publication bias was evaluated. Funnel plots for both ML-based and CRS models showed asymmetry, prompting the use of the trim-and-fill method (Multimedia Appendix 5). After adjustment, pooled AUROC values slightly increased in both groups (ML-based models: 0.88 to 0.90; CRS models: 0.80 to 0.83). However, Egger’s regression test and Begg’s rank correlation test revealed no statistically significant evidence of publication bias for either ML-based models (Egger’s test: *P*=.17; Begg’s test: *P*=.32) or CRS models (Egger’s test: *P*=.53; Begg’s test: *P*=.63).

## Discussion

### Principal Findings

In this review, we appraised studies that assessed the performance of ML-based models compared with CRS for MACCEs prediction after PCI in patients with AMI, using data from 89,702 patients across 10 retrospective studies conducted between 2017 and 2024. Our findings confirmed that ML algorithms outperform CRS methods, such as GRACE and TIMI, in predicting MACCEs risk. Notably, a meta-analysis of 4 studies demonstrated that ML-based models outperform CRS in predicting the mortality risk of AMI patients who had undergone PCI.

ML-based prediction models have gained attention as powerful tools that overcome the limitations of CRS by identifying subtle patterns within complex, multidimensional datasets [6,34]. Predictive models based on CRS rely on fixed assumptions and require prior variable selection, potentially leading to information loss from electronic health records [26]. In other words, CRS may not adequately incorporate real-time data, including treatments and medication administration and may fail to identify changes in patient’s health status after hospital discharge [28,29], potentially resulting in lower accuracy or poorer performance compared to ML-based models. ML does not require preselection because less significant variables are inherently eliminated during model fitting. Additionally, it continuously improves prediction accuracy by learning from new data in real time [26,32]. ML-based models often require a larger number of input variables; however, many studies have shown that they outperform CRS models, particularly in time-sensitive prediction contexts [35,36]. This advantage is partly due to their ability to leverage variables measured closer to the outcome, which enhances predictive accuracy and highlights the clinical utility of using longitudinal in-hospital data. Although their superior predictive performance may sometimes stem from the inclusion of more input variables, recent advancements in feature selection and high-dimensional data processing now allow ML-based models to perform well with fewer clinically relevant predictors [37,38]. These techniques help identify and exclude redundant or low-value variables during model training, enabling the development of more parsimonious models that are easier to implement in clinical settings.

Thus, when combined with CRS, ML-based models can serve complementary roles in developing more effective and reliable predictive models [39]. ML algorithms can rapidly analyze increasingly large datasets, identifying patterns and trends that may not be immediately evident to clinicians. This capability generates opportunities for earlier intervention in situations where CRS may be insufficient [40]. Rather than replacing traditional risk scores, ML-based models can act as dynamic, real-time decision-support tools that complement static risk calculators [35]. This integrated approach may improve clinical decision-making in complex scenarios—for example, in patients with prolonged hospital stays or evolving risk profiles.

Our review specifically found that age, SBP, DBP, MAP, heart rate, creatinine and glucose levels, Killip class, beta-blocker and statin use, and out-of-hospital cardiac arrest were linked to a higher risk of mortality after PCI in patients with AMI, as well as with MACCEs prediction. Among CRS, these variables were primarily components of the GRACE score [41]. This finding suggests that incorporating CRS into ML-based prediction models may be beneficial for identifying high-risk groups. Moreover, age, SBP, and Killip class were among the top-ranked predictors of mortality in patients with AMI who had undergone PCI. These 3 variables can be easily used to predict MACCEs risk after PCI, even before hospital discharge.

Despite the high accuracy and superior discriminative performance of ML-based models in predicting MACCEs, their generalizability remains limited. All included studies were retrospective, and only 2 studies conducted external validation [26,30]. Moreover, 7 of the 10 studies were conducted in Asian countries—including 3 from Korea [25-27]—and relied on registry-based data that may not fully reflect real-world clinical settings. Regional differences in cardiovascular risk profiles may further limit external validity [40]. For example, Sim and Jeong [42] reported that the risk factors for AMI in Korean patients differ from those in Western populations. This geographic concentration introduces potential biases, reducing the applicability of findings to Western or ethnically diverse populations. In addition, the lack of prospective cohort studies limits our ability to evaluate the real-time clinical utility and temporal robustness of ML-based predictions. To address these limitations, future research should prioritize multicenter, prospective studies across diverse populations to improve the generalizability and clinical relevance of ML-based models.

One key barrier to the clinical implementation of ML-based models is the lack of transparency in their decision-making processes—a challenge often described as the “black box” problem [43,44]. This opacity generates concerns among clinicians, who must be able to understand and trust a model’s reasoning before applying it confidently in patient care. Given these concerns, explainable artificial intelligence—such as SHapley Additive exPlanations—is becoming increasingly important [45,46]. These techniques aim to produce ML-based models that are not only effective but also interpretable and trustworthy in clinical settings [46]. By integrating explainable artificial intelligence methods into decision support tools, it is possible to bridge the gap between technical performance and clinical usability—enhancing both clinician confidence and real-world reliability.

This review found that studies on MACCEs after PCI primarily focused on 30-day or 1-year mortality. The 1-year incidence of MACCEs after primary PCI was 10.8%, with most events (73.6%) occurring between 6 months and 1 year postdischarge [47]. Additionally, cardiac deaths accounted for 50% of overall mortality between 6 months and 2 years after AMI [48]. Accordingly, further studies are needed to assess the occurrence of MACCEs over extended follow-up periods to enhance the accuracy of predictive risk models [49].

No significant difference was observed between ML-based models and CRS in predicting 30-day mortality. However, ML-based models consistently outperformed CRS for predicting in-hospital mortality. Although the performance of ML-based models slightly decreased when predicting 30-day mortality, their ability to predict long-term outcomes, particularly 1-year mortality, remained significantly superior to that of CRS. These findings suggest that although the predictive accuracy of ML-based models may slightly decline over longer prediction periods, they continue to offer a distinct advantage in forecasting long-term outcomes. Therefore, future research should incorporate longitudinal data and standardized validation methods to further strengthen the predictive accuracy and clinical applicability of ML-based models.

Substantial heterogeneity was observed across the included studies, aligning with previous findings on the complexity of ML-based prediction models [50]. The meta-regression analysis indicated that smaller sample sizes were significantly associated with higher AUROC values, suggesting possible overestimation due to internal validation or overfitting [51]. However, even after accounting for sample size as a moderator, much of the heterogeneity remained unexplained, pointing to the likely influence of unmeasured factors such as feature engineering, data preprocessing, or institutional differences [50,52]. Future studies should leverage multicenter datasets representing diverse demographic and clinical profiles and conduct prospective external validation across institutions to evaluate real-world generalizability.

Regarding the overall risk of bias assessed using the PROBAST checklist and CHARMS, most studies included in this review had a low risk of bias. However, only 4 studies met over 70% of the TRIPOD + AI criteria, indicating a need for improved adherence to established guidelines while developing predictive models. Consequently, future studies must adhere to rigorous methodological standards to ensure the validity of predictive models for MACCEs after PCI in patients with AMI. Furthermore, enabling data and code sharing during model development may enhance transparency and allow for independent validation of results.

### Implications for Practice and Research

Our review highlights the superior performance of ML-based models in MACCEs prediction after PCI in patients with AMI compared with CRS. Several common predictors of MACCEs or mortality identified in both ML and CRS in this review may help researchers develop more accurate prediction models. However, most common predictors are limited to nonmodifiable clinical characteristics. Therefore, using longitudinal data, modifiable factors, including psychosocial and behavioral variables, should be incorporated into prediction models for MACCEs after PCI in patients with AMI. Health care professionals should understand the advantages and limitations of ML algorithms and CRS before applying them in clinical practice.

### Limitations

This study has certain limitations. This review focused solely on identifying predictors of MACCEs after PCI in patients with AMI. Therefore, our results may not be generalizable to other populations. Despite conducting a comprehensive search across numerous databases to mitigate publication bias, residual bias may still be present, as the analysis was limited to studies published in peer-reviewed journals. Additionally, publication bias may have influenced the results, as studies showing superior ML performance were more likely to be published. Furthermore, all studies in this review were retrospective and had not been performed in clinical practice, posing a significant limitation to their clinical utility. Prospective studies using diverse datasets are needed to ascertain the clinical utility of predictive models and compare their performance with that of clinicians. Additionally, studies included in this review focused on structured data. Therefore, future studies should focus on collecting and integrating unstructured and structured data to develop more accurate risk prediction models.

Lastly, although a meta-regression was conducted to assess the effect of sample size on model performance, it was not feasible to explore all potential sources of heterogeneity due to limitations in the data reported by the included studies. For instance, detailed information on predictor selection strategies and institutional-level variations was often insufficient or inconsistently reported, making comprehensive moderator analysis challenging. Future studies with more standardized reporting may allow for a more nuanced exploration of heterogeneity.

### Conclusions

Our findings indicate that ML algorithms outperform CRS in MACCEs prediction after PCI in patients with AMI. Our review suggests that integrating ML-based models with CRS may improve the precise identification of high-risk patients. Future studies should improve generalizability by including diverse populations and validating ML performance across various ethnicities, age groups, and disease profiles while considering CRS.

## Supplementary material

10.2196/76215 Multimedia Appendix 1 Literature search strategy.

10.2196/76215 Multimedia Appendix 2 Critical appraisal.

10.2196/76215 Multimedia Appendix 3 Importance of variables.

10.2196/76215 Multimedia Appendix 4 Histogram.

10.2196/76215 Multimedia Appendix 5 Funnel plot.

10.2196/76215 Checklist 1 PRISMA checklist.

## Abbreviations

AI: Transparent Reporting of a Multivariable Prediction Model for Individual Prognosis or Diagnosis + Artificial Intelligence
AMI: acute myocardial infarction
AUROC: area under the receiver operating characteristic curve
CHARMS: Checklist for Critical Appraisal and Data Extraction for Systematic Reviews of Prediction Modelling Studies
GRACE: Global Registry of Acute Coronary Events
MACCEs: major adverse cardiovascular and cerebrovascular events
MeSH: Medical Subject Headings
ML: machine learning
PCI: percutaneous coronary interventions
PRISMA: Preferred Reporting Items for Systematic Reviews and Meta-Analyses
PROBAST: Prediction Model Risk of Bias Assessment Tool
PROSPERO: International Prospective Register of Systematic Reviews
TIMI: Thrombolysis in Myocardial Infarction
