# Supplementary material for: Comparing the Performance of Machine Learning Models and Conventional Risk Scores for Predicting Major Adverse Cardiovascular Cerebrovascular Events After Percutaneous Coronary Intervention in Patients With Acute Myocardial Infarction: Systematic Review and Meta-Analysis
Source: J Med Internet Res. 2025 Jul 18;27:e76215. doi: 10.2196/76215 (PMC12295455; doi:10.2196/76215)
Supplement: Multimedia Appendix 1 [file jmir-v27-e76215-s001.docx]

**Multimedia Appendix**. Literature search strategy

| **Database** | **Search No.** | **Search strategy** | **Articles retrieved** |
| --- | --- | --- | --- |
| PubMed | #1 | (((((((((((((((((((Acute Coronary Syndrome[Title/Abstract]) OR (ACS[Title/Abstract])) OR (Coronary Artery Disease[Title/Abstract])) OR (CAD[Title/Abstract])) OR (Heart Attack[Title/Abstract])) OR (ST elevation myocardial infarction[Title/Abstract])) OR (STEMI[Title/Abstract])) OR (non-ST segment elevation acute coronary syndrome[Title/Abstract])) OR (NSTEMI[Title/Abstract])) OR (Unstable Angina[Title/Abstract])) OR (UA[Title/Abstract])) OR (Myocardial Infarction[Title/Abstract])) OR (MI[Title/Abstract])) OR (Ischemic Heart Disease[Title/Abstract])) OR (IHD[Title/Abstract])) OR (Acute Coronary Syndrome[Mesh])) OR (Angina, Unstable [Mesh])) OR (Coronary Artery Disease[Mesh])) OR (Myocardial Infarction[Mesh])) | 104,647 |
|  | #2 | (((((((Percutaneous Coronary Intervention [Title/Abstract])) OR (PCI[Title/Abstract])) OR (stent [Title/Abstract])) OR (revascularization [Title/Abstract])) OR (percutaneous transluminal coronary angioplasty [Title/Abstract])) OR (PTCA[Title/Abstract])) OR (Percutaneous Coronary Intervention [MeSH Terms]) | 55,270 |
|  | #3 | ((((((((Artificial Intelligence [Title/Abstract]) OR (AI[Title/Abstract])) OR (Machine Learning [Title/Abstract])) OR (ML[Title/Abstract])) OR (Deep Learning [Title/Abstract])) OR (DL[Title/Abstract])) OR (predic*[Title/Abstract])) OR (risk*[Title/Abstract])) OR (Artificial Intelligence [Mesh]) | 1,176,239 |
|  | #4 | ((((((((((MACE[Title/Abstract]) OR (major adverse cardiovascular event [Title/Abstract])) OR (MACCE[Title/Abstract])) OR (major adverse cardiovascular [Title/Abstract] AND cerebrovascular event [Title/Abstract])) OR (mortality [Title/Abstract])) OR (readmission [Title/Abstract]))) OR (re-admission [Title/Abstract])) OR (mortality [Title/Abstract])) OR (Patient Readmission [Mesh])) OR (Mortality [Mesh]) | 338,416 |
|  |  | Filters: Full text, Humans, English, Adult: 19+ years, from 2010/1/1 - 2024/10/31 |  |
|  |  | #1 AND #2 AND #3 AND #4 | **8,544** |
| EMBASE | #1 | ('acute coronary syndrome':ab,ti OR acs:ab,ti OR 'coronary artery disease':ab,ti OR cad:ab,ti OR 'heart attack':ab,ti OR 'st segment elevation myocardial infarction':ab,ti OR stemi:ab,ti OR 'non st segment elevation acute coronary syndrome':ab,ti OR nstemi:ab,ti OR 'unstable angina pectoris':ab,ti OR ua:ab,ti OR 'heart infarction':ab,ti OR mi:ab,ti OR 'ischemic heart disease':ab,ti OR ihd:ab,ti OR 'coronary artery disease'/exp OR 'heart infarction'/exp OR 'unstable angina pectoris'/exp) AND [humans]/lim AND [english]/lim AND [01-01-2010]/sd NOT [01-11-2024]/sd AND [2010-2024]/py | 533,345 |
|  | #2 | ('percutaneous coronary intervention':ab,ti OR pci:ab,ti OR stent:ab,ti OR revascularization:ab,ti OR 'percutaneous transluminal coronary angioplasty':ab,ti OR ptca:ab,ti OR 'percutaneous coronary intervention'/exp) AND [humans]/lim AND [english]/lim AND [01-01-2010]/sd NOT [01-11-2024]/sd AND [2010-2024]/py | 229,296 |
|  | #3 | ('artificial intelligence':ab,ti OR ai:ab,ti OR 'machine learning':ab,ti OR ml:ab,ti OR 'deep learning':ab,ti OR dl:ab,ti OR predic*:ab,ti OR risk*:ab,ti OR 'artificial intelligence'/exp OR 'machine learning'/exp) AND [humans]/lim AND [english]/lim AND [01-01-2010]/sd NOT [01-11-2024]/sd AND [2010-2024]/py | 4,908,666 |
|  | #4 | ('mace' OR 'major adverse cardiovascular event':ab,ti OR 'macce':ab,ti OR ('major adverse cardiovascular':ab,ti AND 'cerebrovascular event':ab,ti) OR readmission:ab,ti OR 're admission':ab,ti OR mortality:ab,ti OR 'readmission'/exp OR 'mortality'/exp) AND [humans]/lim AND [english]/lim AND [01-01-2010]/sd NOT [01-11-2024]/sd AND [2010-2024]/py | 1,366,350 |
|  |  | [humans]/lim AND [english]/lim AND [01-01-2010]/sd NOT [01-11-2024]/sd AND [2010-2024]/py |  |
|  |  | #1 AND #2 AND #3 AND #4 | **33,439** |
| CINAHL | #1 | (TI (acs or acute coronary syndrome or stemi or nstemi or non-stemi or mi or myocardial infarction ) OR AB ( acs or acute coronary syndrome or stemi or nstemi or non-stemi or mi or myocardial infarction ) OR TI st elevation myocardial infarction OR AB st elevation myocardial infarction OR TI non-ST segment elevation acute coronary syndrome OR AB non-ST segment elevation acute coronary syndrome OR TI ( ischemic heart disease or IHD ) OR AB ( ischemic heart disease or IHD )) | 6,172 |
|  | #2 | (TI (percutaneous coronary intervention or pci ) OR AB ( percutaneous coronary intervention or pci ) OR TI ( revascularization OR AB ( revascularization ) OR TI ( stent ) OR AB (stent) OR TI ( ‘percutaneous transluminal coronary angioplasty’ or PTCA ) OR AB (‘percutaneous transluminal coronary angioplasty’ or PTCA)) | 3,544 |
|  | #3 | (MH "Artificial Intelligence") OR (TI "Artificial Intelligence" OR AB "Artificial Intelligence") OR (TI "AI" OR AB "AI") OR (MH "Machine Learning") OR (TI "Machine Learning" OR AB "Machine Learning") OR (TI "ML" OR AB "ML") OR (MH "Deep Learning") OR (TI "Deep Learning" OR AB "Deep Learning") OR (TI "DL" OR AB "DL") OR (TI "predic*" OR AB "predic*") OR (TI "risk*" OR AB "risk*")) | 120,916 |
|  | #4 | (TI "MACE" OR AB "MACE") OR (TI "major adverse cardiovascular event" OR AB "major adverse cardiovascular event") OR (TI "MACCE" OR AB "MACCE") OR (TI "major adverse cardiovascular and cerebrovascular event" OR AB "major adverse cardiovascular and cerebrovascular event") OR (TI "readmission" OR AB "readmission") OR (TI "mortality" OR AB "mortality") | 24,422 |
|  |  | S1 AND S2 AND S3 AND S4 | **496** |
| Web of Science | #1 | (((((((((((((((((((((((((((((((TI=(Acute Coronary Syndrome)) OR AB=(Acute Coronary Syndrome))) OR TI=(ACS)) OR AB=(ACS)) OR AU=(Coronary Artery Disease)) OR AB=(Coronary Artery Disease)) OR TI=(CAD)) OR AB=(CAD)) OR TI=(Heart Attack)) OR AB=(Heart Attack)) OR TI=(ST elevation myocardial infarction)) OR AB=(ST elevation myocardial infarction)) OR TI=(STEMI)) OR AB=(STEMI)) OR TI=(non-ST segment elevation acute coronary syndrome)) OR AB=(non-ST segment elevation acute coronary syndrome)) OR TI=(NSTEMI)) OR AB=(NSTEMI)) OR TI=(Unstable Angina)) OR AB=(Unstable Angina)) OR TI=(UA)) OR AB=(UA)) OR TI=(Myocardial Infarction)) OR AB=(Myocardial Infarction)) OR TI=(MI)) OR AB=(MI)) OR TI=(Ischemic Heart Disease)) OR AB=(Ischemic Heart Disease)) OR TI=(IHD)) OR AB=(IHD)) | 318,751 |
|  | #2 | ((((((((((((TI=(Percutaneous Coronary Intervention)) OR AB=(Percutaneous Coronary Intervention)) OR TI=(PCI)) OR AB=(PCI)) OR TI=(stent)) OR AB=(stent)) OR TI=(revascularization)) OR AB=(revascularization)) OR TI=(percutaneous transluminal coronary angioplasty)) OR AB=(percutaneous transluminal coronary angioplasty)) OR TI=(PTCA)) OR AB=(PTCA)) | 131,760 |
|  | #3 | TI=(Artificial Intelligence) OR AB=(Artificial Intelligence) OR TI=(AI) OR AB=(AI) OR TI=(Machine Learning) OR AB=(Machine Learning) OR TI=(ML) OR AB=(ML) OR TI=(Deep Learning) OR AB=(Deep Learning) OR TI=(DL) OR AB=(DL) OR TI=(predic*) OR AB=(predic*) OR TI=(risk* ) OR AB=(risk* ) | 6,330,442 |
|  | #4 | TI=(MACE) OR AB=(MACE) OR TI=(major adverse cardiovascular event) OR AB=(major adverse cardiovascular event) OR TI=(MACCE) OR AB=(MACCE) OR TI=(major adverse cardiovascular and cerebrovascular event) OR AB=(major adverse cardiovascular and cerebrovascular event) OR TI=(readmission) OR AB=(readmission) OR TI=(mortality) OR AB=(mortality) | 780,214 |
|  |  | #1 AND #2 AND #3 AND #4 | **12,683** |
| SCOPUS | #1 | TITLE-ABS-KEY ( acute AND coronary AND syndrome ) OR TITLE-ABS-KEY ( acs ) OR TITLE-ABS-KEY ( coronary AND artery AND disease ) OR TITLE-ABS-KEY ( cad ) OR TITLE-ABS-KEY ( heart AND attack ) OR TITLE-ABS-KEY ( st AND elevation AND myocardial AND infarction ) OR TITLE-ABS-KEY ( stemi ) OR TITLE-ABS-KEY ( non-st AND segment AND elevation AND acute AND coronary AND syndrome ) OR TITLE-ABS-KEY ( nstemi ) OR TITLE-ABS-KEY ( unstable AND angina ) OR TITLE-ABS-KEY ( ua ) OR TITLE-ABS-KEY ( myocardial AND infarction ) OR TITLE-ABS-KEY ( mi ) OR TITLE-ABS-KEY ( ischemic AND heart AND disease ) OR TITLE-ABS-KEY ( ihd ) AND PUBYEAR > 2009 AND PUBYEAR < 2025 AND ( LIMIT-TO ( LANGUAGE , "English" ) ) AND ( LIMIT-TO ( EXACTKEYWORD , "Humans" ) ) | 296,412 |
|  | #2 | TITLE-ABS-KEY (Percutaneous Coronary Intervention) OR TITLE-ABS-KEY(PCI) OR TITLE-ABS-KEY(stent) OR TITLE-ABS-KEY(stent) OR TITLE-ABS-KEY(percutaneous transluminal coronary angioplasty) OR TITLE-ABS-KEY(percutaneous transluminal coronary angioplasty) OR TITLE-ABS-KEY(PTCA) OR TITLE-ABS-KEY(PTCA) AND PUBYEAR > 2009 AND PUBYEAR < 2025 AND ( LIMIT-TO ( LANGUAGE,"English" ) ) AND ( LIMIT-TO ( EXACTKEYWORD,"Humans" ) ) | 113,885 |
|  | #3 | ( TITLE-ABS-KEY ( artificial AND intelligence ) OR TITLE-ABS-KEY ( ai ) OR TITLE-ABS-KEY ( machine AND learning ) OR TITLE-ABS-KEY ( ml ) OR TITLE-ABS-KEY ( deep AND learning ) OR TITLE-ABS-KEY ( dl ) OR TITLE-ABS-KEY ( predic* ) OR TITLE-ABS-KEY ( risk* ) ) AND PUBYEAR > 2009 AND PUBYEAR < 2025 AND ( LIMIT-TO ( LANGUAGE , "English" ) ) AND ( LIMIT-TO ( EXACTKEYWORD , "Humans" ) ) | 2,854,800 |
|  | #4 | ( TITLE-ABS-KEY ( mace ) OR TITLE-ABS-KEY ( major AND adverse AND cardiovascular AND event ) OR TITLE-ABS-KEY ( macce ) OR TITLE-ABS-KEY ( major AND adverse AND cardiovascular AND cerebrovascular AND event ) ) AND PUBYEAR > 2009 AND PUBYEAR < 2025 AND ( LIMIT-TO ( LANGUAGE , "English" ) ) AND ( LIMIT-TO ( EXACTKEYWORD , "Humans" ) ) | 32,883 |
|  |  | #1 AND #2 AND #3 AND #4 | 8,249 |
| Cochrane CENTRAL | #1 | (("Acute Coronary Syndrome":ti,ab,kw) OR ("ACS":ti,ab,kw) OR ("Coronary Artery Disease":ti,ab,kw) OR ("CAD":ti,ab,kw) OR ("Heart Attack":ti,ab,kw) OR ("ST elevation myocardial infarction":ti,ab,kw) OR ("STEMI":ti,ab,kw) OR ("non-ST segment elevation acute coronary syndrome":ti,ab,kw) OR ("NSTEMI":ti,ab,kw) OR ("Unstable Angina":ti,ab,kw) OR ("UA":ti,ab,kw) OR ("Myocardial Infarction":ti,ab,kw) OR ("MI":ti,ab,kw) OR ("Ischemic Heart Disease":ti,ab,kw) OR ("IHD":ti,ab,kw) OR ("Percutaneous Coronary Intervention":ti,ab,kw) OR ("PCI":ti,ab,kw) OR (MeSH descriptor: [Acute Coronary Syndrome] explode all trees) OR (MeSH descriptor: [Angina, Unstable] explode all trees) OR (MeSH descriptor: [Coronary Artery Disease] explode all trees) OR (MeSH descriptor: [Myocardial Infarction] explode all trees)) | 62,788 |
|  | #2 | ("Percutaneous Coronary Intervention":ti,ab,kw) OR ("PCI":ti,ab,kw) OR ("stent":ti,ab,kw) OR ("revascularization":ti,ab,kw) OR ("percutaneous transluminal coronary angioplasty":ti,ab,kw) OR ("PTCA":ti,ab,kw) OR (“Percutaneous Coronary Intervention” explode all trees) | 29,289 |
|  | #3 | (("Artificial Intelligence":ti,ab,kw) OR ("AI":ti,ab,kw) OR ("Machine Learning":ti,ab,kw) OR ("ML":ti,ab,kw) OR ("Deep Learning":ti,ab,kw) OR ("DL":ti,ab,kw) OR ("predic*":ti,ab,kw) OR ("risk*":ti,ab,kw) OR (MeSH descriptor: [Artificial Intelligence] explode all trees)) | 422,419 |
|  | #4 | (MACE):ti,ab,kw OR (major adverse cardiovascular event):ti,ab,kw OR (MACCE):ti,ab,kw OR (major adverse cardiovascular and cerebrovascular event):ti,ab,kw OR (readmission):ti,ab,kw OR (mortality):ti,ab,kw OR ("mortality" explode all trees) | 108,316 |
|  |  | #1 AND #2 AND #3 AND #4(Cochrane Reviews:76) | **5,276** |
| ACM | #1 | Title:(Acute Coronary Syndrome) OR Abstract:(Acute Coronary Syndrome) OR Title:(ACS) OR Abstract:(ACS) OR Title:(Coronary Artery Disease) OR Abstract:(Coronary Artery Disease) OR Title:(CAD) OR Abstract:(CAD) OR Title:(Heart Attack) OR Abstract:(Heart Attack) OR Title:(ST elevation myocardial infarction) OR Abstract:(ST elevation myocardial infarction) OR Title:(STEMI) OR Abstract:(STEMI) OR Title:(non-ST segment elevation acute coronary syndrome) OR Abstract:(non-ST segment elevation acute coronary syndrome) OR Title:(NSTEMI) OR Abstract:(NSTEMI) OR Title:(Unstable Angina) OR Abstract:(Unstable Angina) OR Title:(UA) OR Abstract:(UA) OR Title:(Myocardial Infarction) OR Abstract:(Myocardial Infarction) OR Title:(MI) OR Abstract:(MI) OR Title:(Ischemic Heart Disease) OR Abstract:(Ischemic Heart Disease) OR Title:(IHD) OR Abstract:(IHD) OR Title: (percutaneous coronary intervention) OR Abstract: (percutaneous coronary intervention) OR Title: (PCI) OR Abstract: (PCI) OR Title: (stent) OR Abstract: (stent) OR Title: (revascularization) OR Abstract: (revascularization) OR Title: (percutaneous transluminal coronary angioplasty) OR Abstract: (percutaneous transluminal coronary angioplasty) OR Title: (PTCA) OR Abstract: (PTCA) | 22,119 |
|  | #2 | Title:(Artificial Intelligence) OR Abstract:(Artificial Intelligence) OR Title:(AI) OR Abstract:(AI) OR Title:(Machine Learning) OR Abstract:(Machine Learning) OR Title:(ML) OR Abstract:(ML) OR Title:(Deep Learning) OR Abstract:(Deep Learning) OR Title:(DL) OR Abstract:(DL) OR Title:(predic*) OR Abstract:(predic*) OR Title:(risk* ) OR Abstract:(risk* ) | 89,386 |
|  | #3 | Title:(MACE) OR Abstract:(MACE) OR Title:(major adverse cardiovascular event) OR Abstract:(major adverse cardiovascular event) OR Title:(MACCE) OR Abstract:(MACCE) OR Title:(major adverse cardiovascular and cerebrovascular event) OR Abstract:(major adverse cardiovascular and cerebrovascular event) OR Title:(readmission) OR Abstract:(readmission) OR Title:(mortality) OR Abstract:(mortality) | 560 |
|  |  | 2010/01-2024/10 |  |
|  |  | #1 AND #2 AND #3 | **17** |
| Google Scholar |  | allintitle:(“Acute Coronary Syndrome” OR “Coronary Artery Disease” OR “Heart Attack” OR “ST elevation myocardial infarction” OR “STEMI” OR “non-ST segment elevation acute coronary syndrome” OR “NSTEMI” OR “Unstable Angina” OR “Myocardial Infarction” OR “Ischemic Heart Disease”) AND (“percutaneous coronary intervention” OR “PCI” OR “stent” OR “revascularization” OR “percutaneous transluminal coronary angioplasty” OR “PTCA”) AND (“Artificial Intelligence” OR “Machine Learning” OR “Deep Learning” OR “predic*” OR “risk*”) AND (“MACE” OR “major adverse cardiovascular event” OR “MACCE” OR “major adverse cardiovascular and cerebrovascular event” OR “readmission” OR “mortality”) |  |
|  |  | 2010-2024/english | **5,580** |
| IEEE |  | ("Publication Title":“Acute Coronary Syndrome” OR "Publication Title":“ACS” OR "Publication Title":“Coronary Artery Disease” OR "Publication Title":“CAD” OR "Publication Title":“Heart Attack” OR "Publication Title":“ST elevation myocardial infarction” OR "Publication Title":“STEMI” OR "Publication Title":“non-ST segment elevation acute coronary syndrome” OR "Publication Title":“NSTEMI” OR "Publication Title":“Unstable Angina” OR "Publication Title":“UA” OR "Publication Title":“Myocardial Infarction” OR "Publication Title":“MI” OR "Publication Title":“Ischemic Heart Disease” OR "Publication Title":“IHD”) OR ("Abstract":“Acute Coronary Syndrome” OR "Abstract":“ACS” OR "Abstract":“Coronary Artery Disease” OR "Abstract":“CAD” OR "Abstract":“Heart Attack” OR "Abstract":“ST elevation myocardial infarction” OR "Abstract":“STEMI” OR "Abstract":“non-ST segment elevation acute coronary syndrome” OR "Abstract":“NSTEMI” OR "Abstract":“Unstable Angina” OR "Abstract":“UA” OR "Abstract":“Myocardial Infarction” OR "Abstract":“MI” OR "Abstract":“Ischemic Heart Disease” OR "Abstract":“IHD”) AND ("Publication Title":“percutaneous coronary intervention” OR "Publication Title":“PCI” OR "Publication Title":“stent” OR "Publication Title": “revascularization” OR "Publication Title":“percutaneous transluminal coronary angioplasty” OR "Publication Title":“PTCA”) OR ("Abstract":“percutaneous coronary intervention” OR "Abstract":“PCI” OR "Abstract":“stent” OR "Abstract": “revascularization” OR "Abstract":“percutaneous transluminal coronary angioplasty” OR "Abstract":“PTCA”) AND ("Publication Title":“Artificial Intelligence” OR "Publication Title":“AI” OR "Publication Title":“Machine Learning” OR "Publication Title":“ML” OR "Publication Title":“Deep Learning” OR "Publication Title":“DL” OR "Publication Title":“predic*” OR "Publication Title":“risk*”) OR ("Abstract":“Artificial Intelligence” OR "Abstract":“AI” OR "Abstract":“Machine Learning” OR "Abstract":“ML” OR "Abstract":“Deep Learning” OR "Abstract":“DL” OR "Abstract":“predic*” OR "Abstract":“risk*”) AND ("Publication Title":“MACE” OR "Publication Title":“major adverse cardiovascular event” OR "Publication Title":“MACCE” OR "Publication Title":“major adverse cardiovascular and cerebrovascular event” OR "Publication Title":“readmission” OR "Publication Title":“mortality”) OR ("Abstract":“MACE” OR "Abstract":“major adverse cardiovascular event” OR "Abstract":“MACCE” OR "Abstract":“major adverse cardiovascular and cerebrovascular event” OR "Abstract":“readmission” OR "Abstract":“mortality”) |  |
|  |  | 2010-2024, Journals AND Early Access Articles | **838** |
