# Supplementary material for: Comparing the Performance of Machine Learning Models and Conventional Risk Scores for Predicting Major Adverse Cardiovascular Cerebrovascular Events After Percutaneous Coronary Intervention in Patients With Acute Myocardial Infarction: Systematic Review and Meta-Analysis
Source: J Med Internet Res. 2025 Jul 18;27:e76215. doi: 10.2196/76215 (PMC12295455; doi:10.2196/76215)
Supplement: Multimedia Appendix 2 [file jmir-v27-e76215-s002.docx]

Multimedia Appendix. Critical appraisal

| 1. PROBAST: Risk of bias and applicability assessment tool | | | | | | | | | |
| --- | --- | --- | --- | --- | --- | --- | --- | --- | --- |
| Study No. | Risk of bias | | | | Applicability | | | Overall | |
|  | Participants | Predictors | Outcome | Analysis | Participants | Predictors | Outcome | Risk of bias | Applicability |
| [23] | + | + | + | - | + | + | + | - | + |
| [24] | + | + | + | + | + | + | + | + | + |
| [25] | + | + | + | + | + | + | + | + | + |
| [26] | + | + | + | + | + | - | - | + | - |
| [27] | + | + | + | + | + | + | + | + | + |
| [28] | + | + | + | + | ? | + | + | + | ? |
| [29] | + | - | + | + | + | - | - | - | - |
| [30] | + | + | - | - | - | + | - | - | - |
| [31] | + | + | + | + | - | + | ? | + | - |
| [32] | + | + | + | + | + | + | + | + | + |

Abbreviations: + =indicates low bias/low concern regarding applicability; - = indicates high bias/ high concern regarding applicability; ? = indicates unclear bias/ unclear concern regarding applicability

2. TRIPOD+AI checklist for reporting quality assessment.

| Study No. | Title | Ab-stract | Introduction | | | | Methods | | | | | | | | | | | | | | |
| --- | --- | --- | --- | --- | --- | --- | --- | --- | --- | --- | --- | --- | --- | --- | --- | --- | --- | --- | --- | --- | --- |
|  | Title | Ab-stract | Background | | | Objec-tives | data | | Participants | | | Data pre-paration | Outcome | | | Predictors | | | Sample size | Missing data | |
|  | 1 (D; E) | 2 (D; E) | 3a (D; E) | 3b (D; E) | 3c (D; E) | 4 (D; E) | 5a (D; E) | 5b (D; E) | 6a (D; E) | 6b (D; E) | 6c (D; E) | 7(D; E) | 8a (D; E) | 8b (D; E) | 8c (D; E) | 9a (D) | 9b (D; E) | 9c (D; E) | 10 (D; E) | 11 (D; E) |  |
| [23] | Y | Y | Y | Y | Y | Y | Y | Y | Y | Y | X | Y | Y | X | Y | Y | Y | X | Y | Y |  |
| [24] | Y | Y | Y | Y | Y | Y | Y | Y | Y | Y | X | Y | Y | X | Y | Y | Y | X | Y | P |  |
| [25] | Y | Y | Y | Y | Y | Y | Y | Y | Y | Y | X | Y | Y | X | Y | Y | Y | X | Y | Y |  |
| [26] | Y | Y | Y | Y | Y | Y | Y | Y | Y | Y | X | Y | Y | X | Y | Y | Y | X | Y | Y |  |
| [27] | Y | Y | Y | Y | Y | Y | Y | Y | Y | Y | X | Y | Y | X | Y | Y | Y | X | Y | Y |  |
| [28] | Y | Y | Y | Y | Y | Y | Y | Y | Y | Y | X | Y | Y | X | Y | Y | Y | X | Y | Y |  |
| [29] | Y | Y | Y | Y | Y | Y | Y | Y | Y | Y | X | Y | Y | X | Y | Y | Y | X | Y | Y |  |
| [30] | Y | Y | Y | Y | Y | Y | Y | Y | Y | Y | X | Y | Y | X | Y | Y | Y | X | Y | P |  |
| [31] | Y | Y | Y | Y | Y | Y | Y | Y | Y | Y | X | Y | Y | X | Y | Y | Y | X | Y | Y |  |
| [32] | Y | Y | Y | Y | Y | Y | Y | Y | Y | Y | X | Y | Y | X | Y | Y | Y | X | Y | Y |  |

Abbreviations: D = items relevant only to the development of a prediction model; E = items relating solely to the evaluation of a prediction model; D; E=items applicable to both the development and evaluation of a prediction model; Y= Yes for reported; P= Poorly reported; N= Not reported; x= not applicable; +, >=70% items reported; -, <=70% items reported

2. TRIPOD+AI checklist for reporting quality assessment. (continued)

| Study No. | Methods | | | | | | | | | | | | Open science | | | | | | |
| --- | --- | --- | --- | --- | --- | --- | --- | --- | --- | --- | --- | --- | --- | --- | --- | --- | --- | --- | --- |
|  | Analytical methods | | | | | | | Class im-balance | Fair-ness | Model output | Training versus  evaluation | Ethical approval | Fund-ing | Conflicts of  interest | Pro-tocol | Registration | Data sharing | Code sharing |  |
|  | 12a (D) | 12b (D) | 12c (D) | 12d (D; E) | 12e (D; E) | 12f (E) | 12g (E) | 13 (D; E) | 14 (D; E) | 15(D) | 16 (D; E) | 17 (D; E) | 18a (D; E) | 18b (D; E) | 18c (D; E) | 18d (D; E) | 18e (D; E) | 18f (D; E) |  |
| [23] | Y | Y | Y | N | Y | Y | Y | N | N | Y | N | Y | Y | Y | N | N | N | N |  |
| [24] | Y | Y | Y | N | Y | Y | Y | N | N | Y | N | N | Y | N | N | N | N | N |  |
| [25] | Y | Y | Y | Y | Y | Y | Y | Y | N | Y | Y | Y | Y | Y | N | N | Y | Y |  |
| [26] | Y | Y | Y | N | Y | Y | Y | N | N | Y | N | N | Y | Y | N | Y | N | N |  |
| [27] | Y | Y | Y | N | Y | Y | Y | Y | N | Y | N | Y | Y | Y | N | Y | Y | N |  |
| [28] | Y | Y | Y | N | Y | Y | Y | Y | N | Y | N | Y | X | Y | N | N | Y | N |  |
| [29] | Y | Y | Y | N | Y | Y | Y | Y | N | Y | Y | N | Y | Y | N | N | N | Y |  |
| [30] | Y | Y | Y | X | Y | Y | Y | N | N | Y | N | Y | N | Y | N | N | Y | N |  |
| [31] | Y | Y | Y | X | Y | X | X | N | N | Y | N | Y | Y | Y | Y | N | Y | N |  |
| [32] | Y | Y | Y | X | Y | Y | Y | N | N | Y | N | Y | Y | Y | Y | N | Y | Y |  |

Abbreviations: D = items relevant only to the development of a prediction model; E = items relating solely to the evaluation of a prediction model; D; E=items applicable to both the development and evaluation of a prediction model; Y= Yes for reported; P= Poorly reported; N= Not reported; x= not applicable; +, >=70% items reported; -, <=70% items reported

2. TRIPOD+AI checklist for reporting quality assessment. (continued)

| Study No. | Patient & Public Involvement | Results | | | | | | | | Discussion | | | | | Quality |
| --- | --- | --- | --- | --- | --- | --- | --- | --- | --- | --- | --- | --- | --- | --- | --- |
|  | Patient & Public  Involvement | Participants | | | Model develop-ment | Model speci-fication | Model per-formance | | Model updating | Inter-pretation | Limi-tations | Usability of the model in the context of current care | | |  |
|  | 19 (D; E) | 20a (D; E) | 20b (D; E) | 20c (D; E) | 21 (D; E) | 22 (D; E) | 23a (D; E) | 23b (D; E) | 24 (D; E) | 25 (D; E) | 26 (D; E) | 27a (D; E) | 27b (D; E) | 27c (D; E) |  |
| [23] | N | Y | Y | N | Y | N | P | N | N | Y | Y | Y | Y | Y | - |
| [24] | N | Y | Y | N | Y | N | P | N | N | Y | Y | N | Y | Y | - |
| [25] | N | Y | Y | N | Y | Y | Y | N | Y | Y | Y | Y | Y | Y | + |
| [26] | N | Y | Y | N | Y | N | P | N | N | Y | Y | Y | Y | Y | - |
| [27] | N | Y | Y | N | Y | N | Y | N | N | Y | Y | Y | Y | Y | + |
| [28] | N | Y | Y | N | Y | N | P | N | N | Y | Y | Y | Y | P | - |
| [29] | N | Y | Y | N | Y | Y | Y | N | Y | Y | Y | Y | Y | Y | + |
| [30] | N | Y | Y | N | Y | N | Y | N | N | Y | Y | Y | Y | P | - |
| [31] | N | Y | Y | X | Y | N | Y | N | X | Y | Y | Y | Y | Y | - |
| [32] | N | Y | Y | N | Y | Y | Y | N | Y | Y | Y | Y | Y | Y | + |

Abbreviations: D = items relevant only to the development of a prediction model; E = items relating solely to the evaluation of a prediction model; D; E=items applicable to both the development and evaluation of a prediction model; Y= Yes for reported; P= Poorly reported; N= Not reported; x= not applicable; +, >=70% items reported; -, <=70% items reported

3. Critical appraisal and data extraction for systematic reviews of prediction modeling studies (CHARMS) checklist evaluations.

| Study No. | Source of data/  participant selection | Outcomes | Candidate  predictors | Sample size/  missing data | Attrition | Model development | Model performance/  evaluation |
| --- | --- | --- | --- | --- | --- | --- | --- |
| [23] | L | L | L | M | L | M | L |
| [24] | L | L | L | L | M | M | L |
| [25] | L | L | M | H | H | M | L |
| [26] | L | L | L | L | M | M | M |
| [27] | L | L | L | L | M | L | M |
| [28] | L | L | L | L | M | M | M |
| [29] | L | L | L | L | L | L | M |
| [30] | L | L | L | H | L | L | L |
| [31] | L | L | L | L | M | M | M |
| [32] | L | L | L | H | H | M | M |

Abbreviations : L= low; M= moderate; H= high risk of bias.
