# Supplementary material for: Comparing the Performance of Machine Learning Models and Conventional Risk Scores for Predicting Major Adverse Cardiovascular Cerebrovascular Events After Percutaneous Coronary Intervention in Patients With Acute Myocardial Infarction: Systematic Review and Meta-Analysis
Source: J Med Internet Res. 2025 Jul 18;27:e76215. doi: 10.2196/76215 (PMC12295455; doi:10.2196/76215)
Supplement: Multimedia Appendix 3 [file jmir-v27-e76215-s003.docx]

Multimedia Appendix. Importance of variables of MACCE in AMI patients undergoing PCI. (N=10)

| Study No. | First author  (year) | Importance of variables | |
| --- | --- | --- | --- |
|  |  | Machine learning–based models | Conventional risk score models |
| [23] | Shouval et al. (2017) | Age, creatinine, Killip at admission, blood pressure (SBP/DBP), glucose level. | Age, weight, smoking status, [medical history](https://www-sciencedirect-com.proxy.cau.ac.kr/topics/medicine-and-dentistry/medical-history), past medical history, drugs at admission, vital signs at admission, [time to treatment](https://www-sciencedirect-com.proxy.cau.ac.kr/topics/medicine-and-dentistry/time-to-treatment), creatinine, [cardiac markers](https://www-sciencedirect-com.proxy.cau.ac.kr/topics/medicine-and-dentistry/cardiac-marker), and ECG finding. |
| [24] | Kim et al.  (2022) | CABG, Statin, History of hypertension, Resuscitation, Left pain, Angiographic findings, Glucose, Family history of HD, 2-vessel, SBP, Pre-TIMI flow, LDL-C, History of DM, PCI, Heart ratio, Complications, Creatinine, Abnormal cardiac enzyme, Pain, History of smoking, Weight, History of IHD, STMI, Age, Maximum TnI, Killip class, NT-pro BNP, Total cholesterol. | Age, ST segment deviation on EKG, Creatinine, Abnormal cardiac enzyme, Killip class, Heart rate, SBP, Cardiac arrest at admission |
| [25] | Kwon et al.  (2019) | Killip class, Age, OHCA, Creatinine, Glucose, SBP, Heart rate, ST elevation, CK-MB, LDL, Gender, CRP, BMI. | Killip class, Age, OHCA, Creatinine, Glucose, SBP, Heart rate, ST elevation, CK-MB, LDL, Gender, CRP, BMI |
| [26] | Sherazi et al.  (2020) | Age >76years, coronary angiogram was not performed in angiographic findings, diuretics, Killip class III, creatinine, LV ejection fraction. | Age 80–89years, Age 70–79years, Killip III, HR <70bpm, Killip IV, Killip II, Creatinine > 2.0 ng/ dL, Killip I, Age <30years |
| [27] | Aziz et al.  (2021) | Age, Race, Smoking status, Hypertension, Diabetes, Family history of premature CVD, Chronic renal disease, Heart rate, SBP, DBP, Killip class, HDL, Fasting blood glucose, Weight, ECG-type bundle branch block, ECG- location lateral lead, Time to treatment, Cardiac catheterization, PCI, ASA, Beta blockers, ACE inhibitor, Statin, Diuretics, Oral hypo glycemic agent, insulin. | Age, Hypertension, Diabetes, Heart rate, SBP, Killip class, Weight, ECG-type bundle branch block, Time to treatment. |
| [28] | Bai et al.  (2021) | ALT, Shock, BNP, Diabetes mellitus, No working days, LDH, WBC, CK-MB, HBDH. | Not reported |
| [29] | Hadanny et al. (2021) | Creatinine (mg/dL), MAP (mmHg), Killip class at admission, Glucose (mmol/L), Age, Total Cholesterol (mg/dL), Hemoglobin (g/dL), Heart Rate (bmp), BMI, Time from Onset to PCI (minutes) | Age, [medical history](https://www-sciencedirect-com.proxy.cau.ac.kr/topics/pharmacology-toxicology-and-pharmaceutical-science/medical-history), vital signs at admission, creatinine, [cardiac markers](https://www-sciencedirect-com.proxy.cau.ac.kr/topics/medicine-and-dentistry/cardiac-marker), and ECG findings. |
| [30] | Fang et al.  (2022) | SBP, DBP, Killip grade II-IV, Urea, LVEF, NT-Pro BNP, IABP | Killip class II-IV, urea nitrogen, LVEF, NT-Pro BNP |
| [31] | Liu et al.  (2024) | Urea, SBP, WBC, Hemoglobin, PLT, Gfr, myoglobin, cystatin, BMI, monocytes, lipa, AST, TG, TC, communication ability, ALT, discharge outcomes, TB, GLU, lactic acid, LDL, CRP, CK, Glycated hemoglobin, CTnT, mode of admission, HDL. | Not reported |
| [32] | Shakhgeldyan et al. (2024), | Age, HR, SBP, AHF T. Killip class, Creatinine, LVEF, NEUT, EOS, PCT, Glucose. | Age, HR, SBP, AHF T. Killip class, Creatinine |

Abbreviations : ACE=angiotensin converting enzyme inhibitor; AF=atrial fibrillation; ASA = acetylsalicylic acid (aspirin); AST=aspartate aminotransferase; ALT=alanine aminotransferase; ARB=angiotensin receptor blocker; ARNI=angiotensin receptor neprilysin inhibitor; APTT = activated partial thromboplastin time; AHF T. Killip class =acute heart failure class according to T. Killip; BMI=body mass index; BNP=B-type natriuretic peptide; BP=blood pressure; BUN=blood urea nitrogen; CABG= coronary artery bypass grafting; CKD= chronic kidney disease; CK =Creatine Kinas; CK-MB = Creatine kinase myoglobin; COPD=chronic obstructive pulmonary disease; CRP=C-reactive protein; CRT-D/P=cardiac resynchronization therapy with defibrillator/pacer function; CTnT =Anti-Cardiac Troponin T; CVD = cardiovascular disease; DBP=diastolic blood pressure; DM=diabetes mellitus; ECG = electrocardiography; EOS= eosinophils; ; Gfr =Glomerular filtration rate; Glu= glucose; Hb= hemoglobin; HBDH =Hydroxybutyrate dehydrogenase; HD= heart disease; HDL=High Density Lipoprotein; HF=heart failure; HR= heart rate; HTN=hypertension; IABP= intra-aortic balloon pump; IHD= ischemic heart disease ;INR= international normalized ratio; LDH=lactate dehydrogenase; LDL-C= low density lipoprotein-cholesterol; LVEF=left ventricular ejection fraction; MAP= Mean Arterial pressure; MI=myocardial infarction; MT=medical therapy; MPAP= mean pulmonary artery pressure; NEUT= neutrophils; NT-pro BNP= N-terminal brain natriuretic peptide; OHCA= out-of-hospital cardiac arrest; PCI=percutaneous coronary intervention; PCT= thrombocrit; PLT=platelets; RBC= erythrocytes; SBP= systolic blood pressure; TB = Tuberculosis; TC = total cholesterol; TG = triglyceride; TH= thrombolysis; TIMI=Thrombolysis in Myocardial Infarction; TnI= troponin-I; TnT= troponin-T; WBC= leukocyt
