# Supplementary material for: Comparing the Performance of Machine Learning Models and Conventional Risk Scores for Predicting Major Adverse Cardiovascular Cerebrovascular Events After Percutaneous Coronary Intervention in Patients With Acute Myocardial Infarction: Systematic Review and Meta-Analysis
Source: J Med Internet Res. 2025 Jul 18;27:e76215. doi: 10.2196/76215 (PMC12295455; doi:10.2196/76215)
Supplement: Multimedia Appendix 4 [file jmir-v27-e76215-s004.docx]

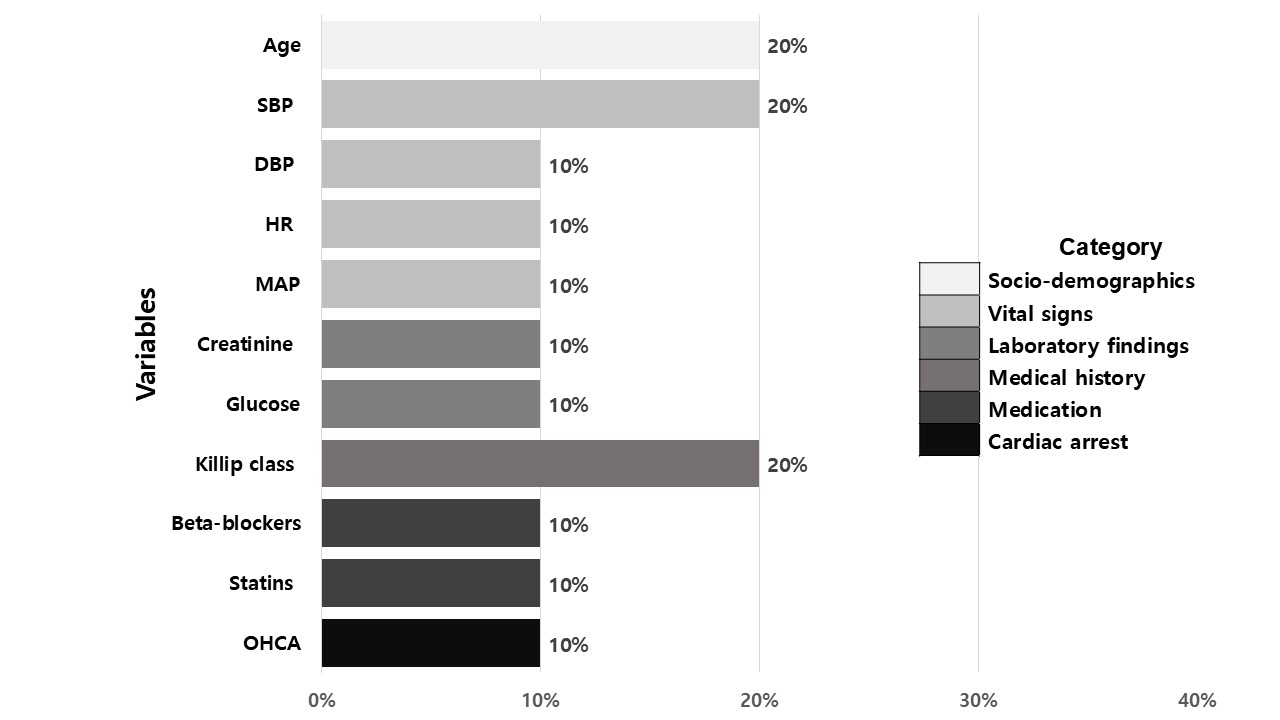


Multimedia Appendix**.** Histogram of common mortality variables reported in individual studies

Abbreviations: DBP=diastolic blood pressure; HR=heart rate; MAP=Mean Arterial Pressure; OHCA=out-of-hospital cardiac arrest; SBP=systolic blood pressure.
