# Supplementary material for: Comparing the Performance of Machine Learning Models and Conventional Risk Scores for Predicting Major Adverse Cardiovascular Cerebrovascular Events After Percutaneous Coronary Intervention in Patients With Acute Myocardial Infarction: Systematic Review and Meta-Analysis
Source: J Med Internet Res. 2025 Jul 18;27:e76215. doi: 10.2196/76215 (PMC12295455; doi:10.2196/76215)

Multimedia Appendix: Contour-enhanced funnel plots for machine learning–based models and conventional risk score models with trim-and-fill method. AUROC: area under the receiver operating characteristic curve; ML: machine learning; CRS: conventional risk score.


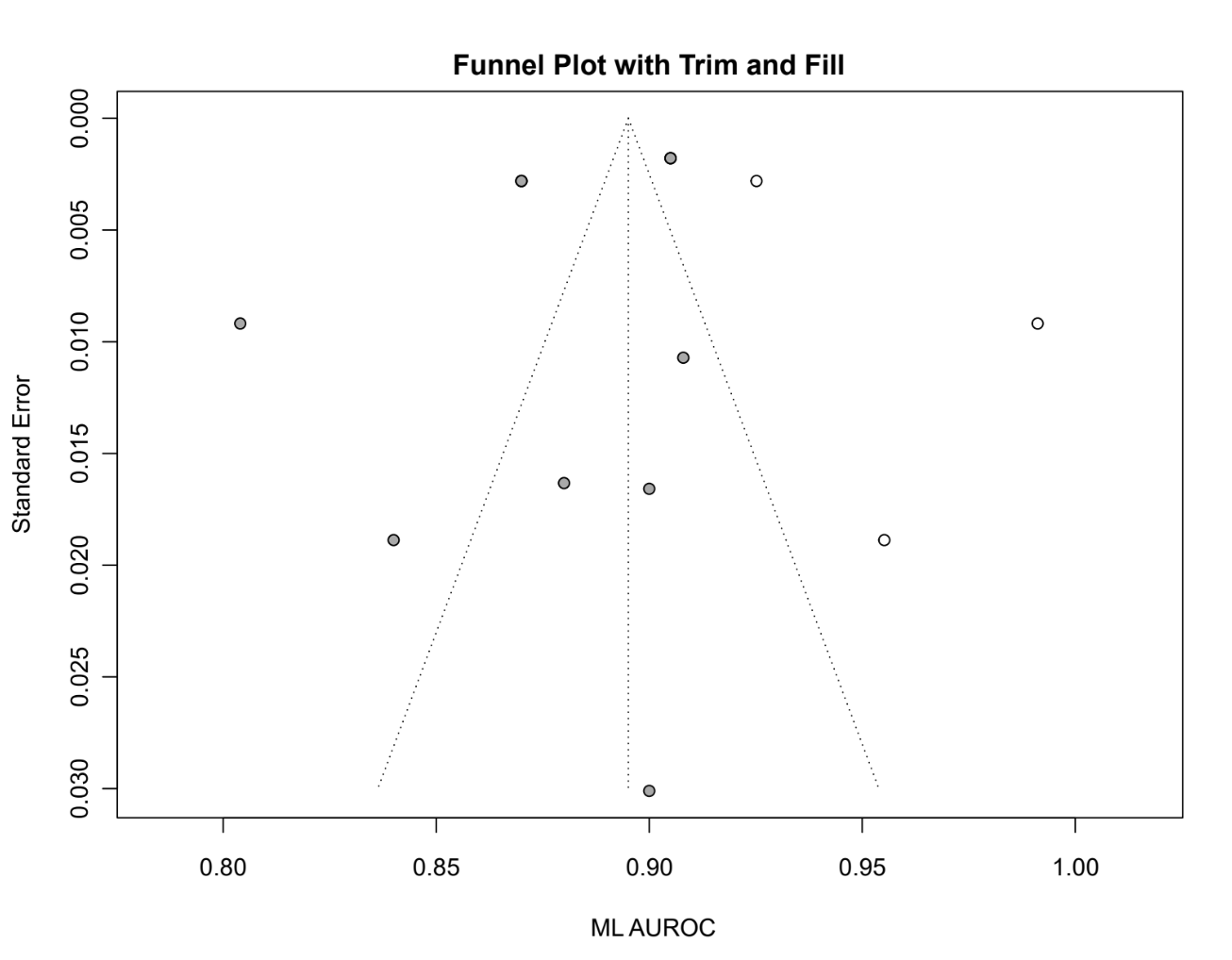

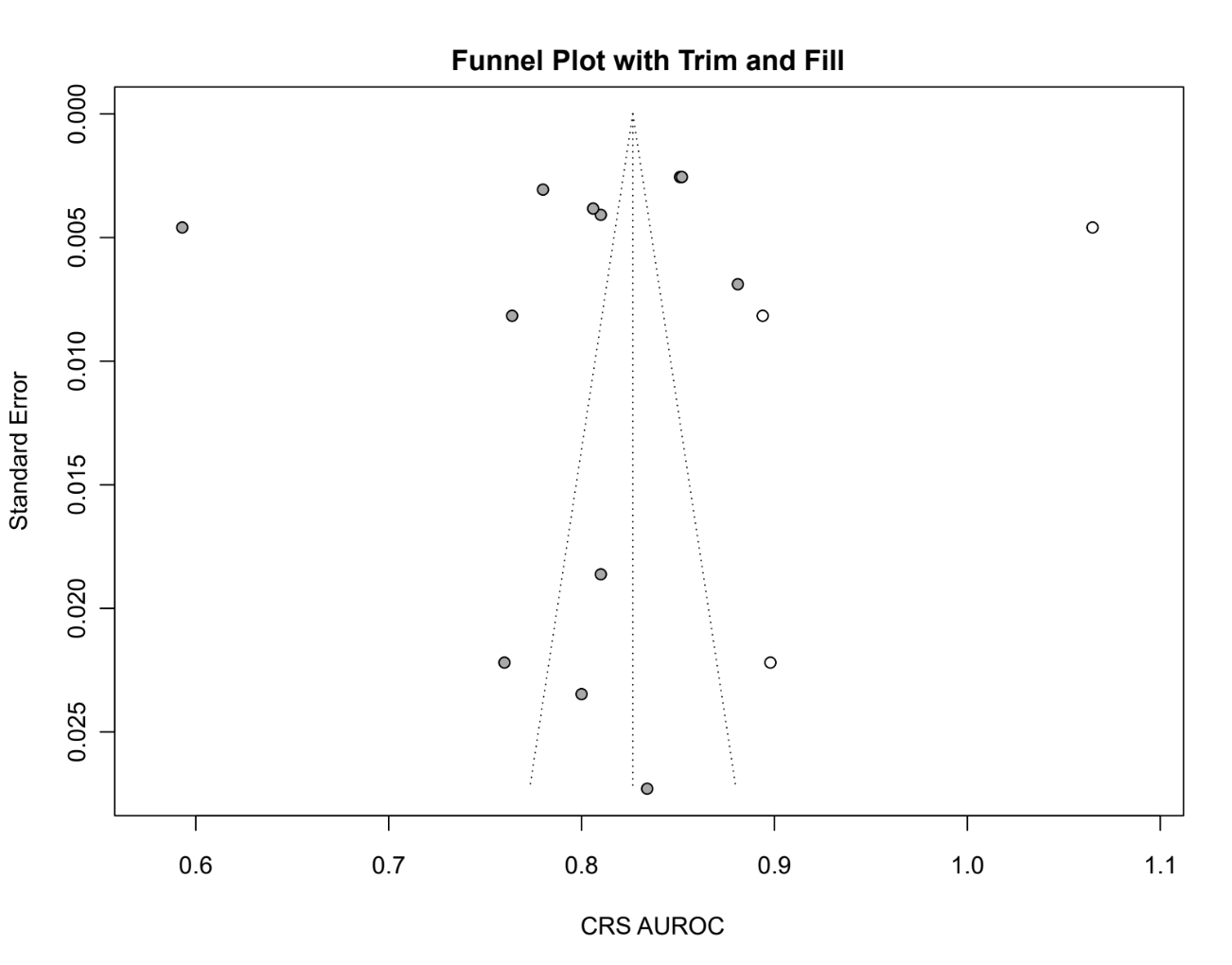

Supplement: Multimedia Appendix 5 [file jmir-v27-e76215-s005.docx]
